# Prevalence and Determinants of Anemia Among Adolescent Girls: A Community-Based Cross-Sectional Study in a Rural Field Practice Area of a Tertiary Hospital, India

**DOI:** 10.7759/cureus.101423

**Published:** 2026-01-13

**Authors:** Dibyanshu Dibyanshu, Mary Moses, Prima Lakra, Rainita R Pise, Vineeta Vineeta, Divya Shubham, Alok Kumar, N B Kasturwar

**Affiliations:** 1 Community and Family Medicine, All India Institute of Medical Sciences, Deoghar, Deoghar, IND; 2 Community Medicine, Mamata Academy of Medical Sciences, Hyderabad, IND; 3 Pathology and Laboratory Medicine, All India Institute of Medical Sciences, Deoghar, Deoghar, IND; 4 Community Medicine, Datta Meghe Medical College, Nagpur, IND; 5 Obstetrics and Gynaecology, All India Institute of Medical Sciences, Deoghar, Deoghar, IND; 6 Obstetrics and Gynecology, Lord Buddha Koshi Medical College and Hospital, Saharsa, IND; 7 Community Medicine, Mahabodhi Medical College, Gaya, IND; 8 Community Medicine, Narendra Kumar Prasadrao (NKP) Salve Institute of Medical Sciences & Research Centre, Nagpur, IND

**Keywords:** adolescent girls, anemia, community-based study, india, menstrual morbidity, rural health

## Abstract

Background

Anemia is a major public health challenge among adolescent girls, particularly in rural India, where nutritional deficiencies, menstrual morbidities, and limited access to preventive services converge. Despite several national-level initiatives aimed at improving adolescent nutritional health, prevalence remains high.

Objectives

This study aimed to estimate the prevalence and severity of anemia and identify its determinants among adolescent girls aged 10-19 years in a rural community of Nagpur, India.

Methods

A community-based cross-sectional study was conducted over one year (December 2014-November 2015) among 600 adolescent girls selected via systematic random sampling at Narendra Kumar Prasadrao (NKP) Salve Institute of Medical Sciences & Research Centre, Nagpur, Maharashtra. Data were collected using a pre-tested semi-structured questionnaire and included sociodemographic factors, menstrual history, anthropometry, and hemoglobin estimation via Sahli’s method. Anemia was classified using World Health Organization guidelines. Statistical analysis was performed using IBM SPSS Statistics for Windows, version 25.0 (IBM Corp., Armonk, NY).

Results

The overall prevalence of anemia was 80% (95% confidence interval (CI): 76.8-83.2%), with 60% mild, 30% moderate, and 10% severe cases. Significant associations were observed between anemia severity and both age (p < 0.001) and menstrual morbidities (p < 0.001), whereas the body mass index showed no significant correlation (p = 0.553). Older adolescents and those with menstrual morbidities exhibited higher anemia severity.

Conclusion

Anemia among rural adolescent girls is alarmingly high and closely linked to reproductive and sociodemographic factors. Interventions must extend beyond iron supplementation to include menstrual health education, maternal literacy promotion, and routine community-based screening.

## Introduction

Adolescence, spanning 10 to 19 years of age, is a pivotal period for physiological, cognitive, and psychosocial development [1]. For girls, this life stage is further complicated by the onset of menarche and increased nutritional demands. Iron requirements rise significantly due to expanded blood volume, muscle mass, and menstrual blood loss. When these demands are unmet, anemia, particularly iron-deficiency anemia, can develop rapidly, compromising adolescent health and future reproductive potential [2].

Anemia remains a pervasive global health issue, affecting approximately 40% of children aged six to 59 months, 37% of pregnant women, and 30% of women between 15 and 49 years. In 2019 alone, anemia accounted for an estimated 50 million years of healthy life lost due to disability, with dietary iron deficiency, hemoglobinopathies such as thalassemia and sickle cell trait, and malaria identified as the principal contributors [3]. Despite several national-level initiatives aimed at improving adolescent nutritional health, the prevalence of anemia remains high. According to the National Family Health Survey-4 (NFHS-4), 53.1% of adolescent girls aged 15-19 years are anemic, a troubling increase from previous estimates, highlighting persistent deficiencies in community outreach, early diagnostic efforts, and the integration of menstrual health within adolescent health frameworks [4]. Anemia in adolescence is not merely a nutritional deficiency; it is a systemic risk factor. It impairs educational performance, work productivity, and immune competence and serves as a precursor to maternal anemia, obstetric complications, and adverse neonatal outcomes. Addressing anemia early is therefore critical, not just for individual well-being but also for public health outcomes across the life course [5].

While several studies have investigated the prevalence of anemia in institutional or urban school-based settings, there remains a paucity of community-based data from rural India, where the burden is often higher. Furthermore, few investigations have explored the complex interplay between anemia and contributory factors such as menstrual morbidities, nutritional status, socioeconomic background, and maternal education. In response to this critical evidence gap, the present study was undertaken with the following objectives: to estimate the prevalence of anemia among adolescent girls aged 10-19 years residing in a rural field practice area of a tertiary care teaching hospital in Nagpur; to determine the severity of anemia based on WHO hemoglobin cut-offs and classify cases as mild, moderate, or severe; and to identify the factors influencing anemia.

## Materials and methods

This community-based cross-sectional study was conducted from December 2014 to November 2015 in the rural field practice area of Narendra Kumar Prasadrao (NKP) Salve Institute of Medical Sciences & Research Centre, a tertiary-care teaching hospital in Nagpur, Maharashtra. This area, affiliated with the Department of Community Medicine of a tertiary care institute in Nagpur, serves an estimated catchment population of approximately 120,000. The area is predominantly agrarian, with low-to-moderate literacy rates and limited access to secondary healthcare facilities. The study population included adolescent girls aged 10-19 years who had attained menarche, were available during visits, and consented to participate. Girls with diagnosed hemoglobinopathies, chronic illness (like cancer), or communication-impairing mental conditions were excluded. The sample size was calculated by taking the prevalence of menstrual morbidities as 42% from a previous study and a relative precision of 10%. The formula of Zpq/d^2 ^was used. A sample size of 560 was calculated (p = 42%, d = 10% of 42), which was rounded up to 600, taking into account the attrition rate. The participants were selected from a sampling frame of 6424 (adolescent girls) via systematic random sampling, with the sampling interval being 11 [6]. NKP Salve Institute of Medical Sciences & Research Centre Institutional Ethics Committee issued approval (no. 2013/32).

Data were collected using a pre-tested, semi-structured questionnaire translated into Marathi and back-translated into English. Face validity was evaluated during pre-testing with 30 adolescent girls from a neighbouring village, and modifications were made to improve clarity and cultural appropriateness. The tool captured sociodemographic data, menstrual history, and socioeconomic status (assessed using the Modified Brahm Govind Prasad scale, December 2014 Consumer Price Index) [7]. 

Before starting fieldwork, data collectors underwent a structured two-day training led by senior faculty members to ensure the quality of data collection. The sessions covered ethical principles in human research, standardized interviewing techniques, anthropometric measurement protocols, hemoglobin estimation procedures, and the importance of maintaining participant confidentiality. Practical components included mock interviews, supervised anthropometric measurements, and hands-on hemoglobin estimation to ensure uniformity in procedures. All instruments, including weighing machines and Sahli’s hemoglobinometers, were calibrated daily to maintain accuracy. Quality control was ensured through random on-site observations by supervisors, daily checks of completed questionnaires, and double data entry to minimize errors.

Hemoglobin was measured on-site using Sahli’s hemoglobinometer. Anemia was defined as Hb <12 g/dL and classified as mild (10-11.9), moderate (7-9.9), or severe (<7 g/dL). Severely anemic girls were referred, and all anemic participants received dietary and menstrual hygiene counseling.

Data collection took place at households, ensuring privacy. Daily reviews ensured data quality. Double data entry was done in Excel and cross-validated. Ethical approval and community permissions were obtained. Informed consent/assent was secured. Statistical analysis was performed using IBM SPSS Statistics for Windows, version 25.0 (released 2017, IBM Corp., Armonk, NY) and Python 3.4.2, with chi-square tests for associations (p ≤ 0.05 considered significant).

## Results

A total of 600 adolescent girls were included in the study. Most participants belonged to the 14-16 years' age group (53%), followed by those aged 17-19 years (43%) and 10-13 years (4%). Over half (54%) of the girls had a high school education, while 38% and 8% were educated up to middle and primary school levels, respectively. Socioeconomic status assessment using the Modified BG Prasad Scale showed that 45% belonged to the middle class, 27% to the upper middle, and 20% to the lower middle class. Mothers of the participants were predominantly educated up to high school (42%), and most were unemployed (65%), as shown in Table 1.

**Table 1:** Sociodemographic profile of the adolescent girls (n = 600)

Variable	Category	Frequency (n)	Percentage (%)
Age group (years)	10–13 (early adolescence)	24	4.0
	14–16 (middle adolescence)	318	53.0
	17–19 (late adolescence)	258	43.0
Religion	Hindu	342	57.0
	Buddhist	174	29.0
	Muslim	72	12.0
	Others	12	2.0
Type of family	Nuclear	558	93.0
	Joint	24	4.0
	Three generation	18	3.0
Education (participant)	Primary school	48	8.0
	Middle school	228	38.0
	High school	324	54.0
Mother’s education	Illiterate	54	9.0
	Primary school	78	13.0
	Middle school	114	19.0
	High school	252	42.0
	Intermediate	66	11.0
	Graduate	36	6.0
Mother’s occupation	Unemployed	390	65.0
	Unskilled worker	126	21.0
	Semi-skilled worker	36	6.0
	Skilled worker	12	2.0
	Clerical/farmer/shop owner	36	6.0
Socioeconomic status	Class I (upper class)	24	4.0
	Class II (upper middle class)	162	27.0
	Class III (middle class)	270	45.0
	Class IV (lower middle class)	120	20.0
	Class V (lower class)	24	4.0

**Figure 1:**
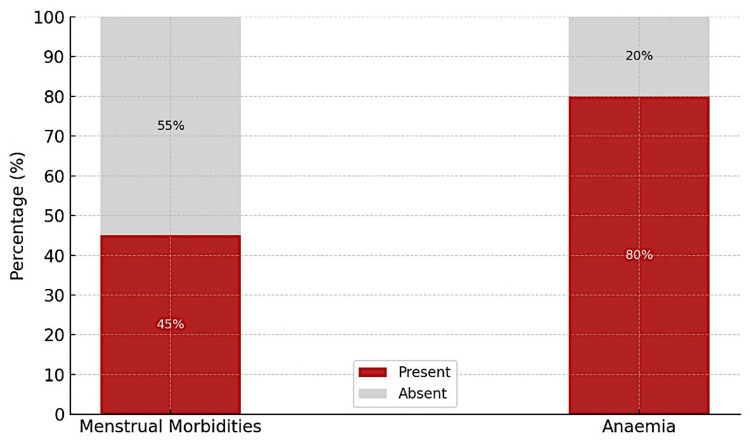
Distribution of participants based on menstrual abnormalities and anemia (n = 600) Image created by the authors with Microsoft Excel version 2019 (Microsoft Corp., USA)

As shown in Figure 1, menstrual morbidities (menorrhagia, oligomenorrhea, dysmenorrhea, hypomenorrhea, and menometrorrhagia) were reported by 45% of the participants, while anemia was present in 80% (95% CI: 76.8-83.2%), underscoring the dual burden of menstrual and hematological health challenges in this population.

**Figure 2:**
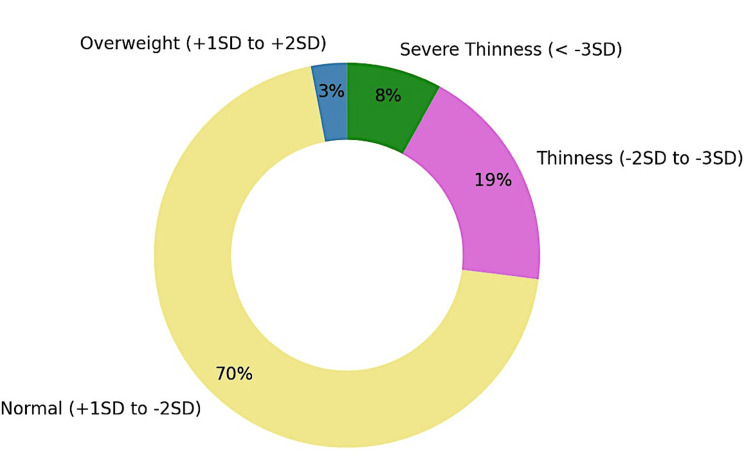
Distribution of participants according to BMI (n = 600) based on the WHO growth standards SD: standard deviation Image created by the authors with Microsoft Excel version 2019 (Microsoft Corp., USA)

Figure 2 demonstrates that the majority of the participants (70%) had a normal BMI according to WHO growth standards, with 19% classified as thin, 8% as severely thin, and 3% as overweight [8].

**Figure 3:**
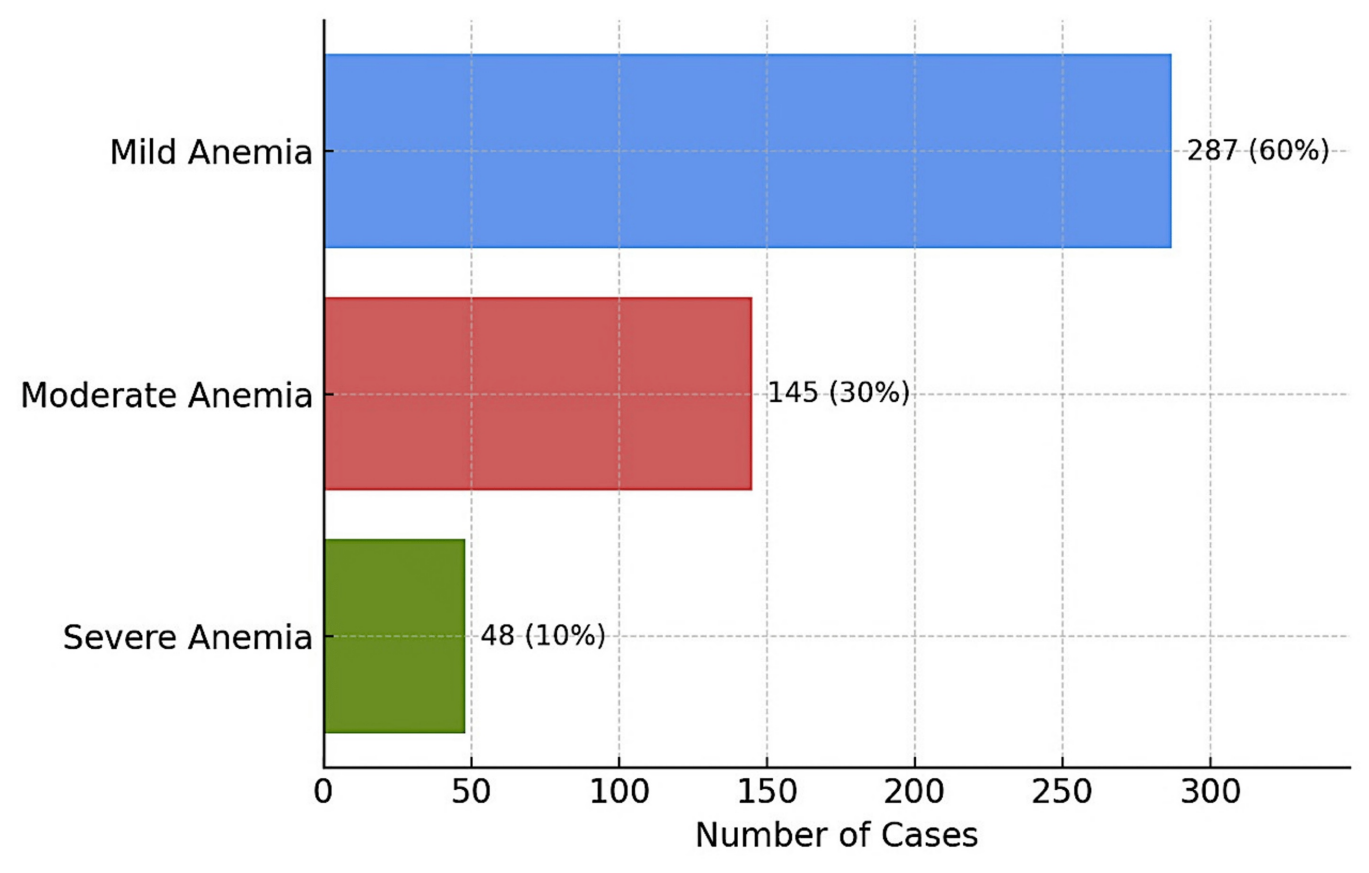
Distribution of participants based on the severity of anemia (n = 480) Image created by the authors with Microsoft Excel version 2019 (Microsoft Corp., USA)

Figure 3 reveals the distribution of anemia severity among the 480 affected participants: 60% had mild anemia, 30% had moderate anemia, and 10% had severe anemia.

Among the 600 adolescent girls studied, 45% of the participants had menstrual morbidities.

Among the 600 adolescent girls studied, those in the 14-16 and 17-19 years' age groups exhibited higher proportions of moderate and severe anemia compared to younger adolescents (p < 0.001). Similarly, girls reporting menstrual morbidities were more likely to experience moderate and severe anemia (45.5%) compared to those without morbidities (21.2%), a difference that was also highly significant (p < 0.001). By contrast, although anemia was more frequent among girls with severe thinness, the association between BMI category and anemia severity did not reach statistical significance (p = 0.553), as shown in Table 2.

**Table 2:** Association of anemia severity with age group, menstrual morbidities, and BMI Z-score (n = 600) *Chi-square test was used to assess the association between anaemia severity and each independent variable. P-values < 0.05 were considered statistically significant.

Variable	Normal n (%)	Mild n (%)	Moderate n (%)	Severe n (%)	Chi-square statistic	p-value*
Age group					28.86	<0.001
10–13 years	6 (25.0%)	18 (75.0%)	0 (0.0%)	0 (0.0%)		
14–16 years	55 (17.3%)	155 (48.7%)	92 (28.9%)	16 (5.0%)		
17–19 years	59 (22.9%)	114 (44.2%)	53 (20.5%)	32 (12.4%)		
Menstrual morbidity					48.27	<0.001
Present	44 (16.3%)	103 (38.1%)	84 (31.1%)	39 (14.4%)		
Absent	76 (23.0%)	184 (55.8%)	61 (18.5%)	9 (2.7%)		
BMI Z-Score					7.82	0.553
Severe thinness (< −3 SD)	14 (28.0%)	18 (36.0%)	14 (28.0%)	4 (8.0%)		
Thinness (−2 to −3 SD)	20 (17.4%)	56 (48.7%)	25 (21.7%)	14 (12.2%)		
Normal (−2 to +1 SD)	82 (19.6%)	205 (48.9%)	103 (24.6%)	29 (6.9%)		
Overweight (+1 to +2 SD)	4 (25.0%)	8 (50.0%)	3 (18.8%)	1 (6.2%)		

## Discussion

This study identified a notably high prevalence of anemia (80%) among adolescent girls residing in the rural field practice area of a tertiary care teaching hospital in Nagpur, a figure substantially exceeding the national average of 53.1% reported in the NFHS-4 for adolescent girls aged 15-19 years [4]. The predominance of mild and moderate anemia (accounting for over 70% of all cases) underscores a pervasive subclinical burden that, while not always symptomatic, poses substantial risks to physical and cognitive development, particularly in populations transitioning into reproductive age. These findings reinforce the persistent and disproportionate impact of anemia in rural India, where health system outreach, nutritional diversity, and access to preventive services remain constrained.

In addition to the overall burden, anemia severity demonstrated statistically significant associations with increasing age and the presence of menstrual morbidities. Older adolescents (aged 17-19 years) exhibited a greater frequency of moderate and severe anemia compared to their younger counterparts, potentially reflecting the cumulative nutritional deficits and elevated iron requirements associated with menarche and pubertal progression. Furthermore, the study found that girls reporting menstrual abnormalities such as menorrhagia, polymenorrhagia, and dysmenorrhea had significantly higher rates of moderate-to-severe anemia, suggesting that menstrual health may be an underrecognized driver of iron depletion in this demographic. Conversely, no significant association was found between BMI and anemia severity, indicating that anthropometric nutritional status alone may be an insufficient proxy for micronutrient adequacy, particularly iron stores. Together, these findings highlight critical physiological, reproductive, and systemic determinants of anemia in rural adolescents, offering essential insights for tailored public health interventions.

This study reports a prevalence of anemia of 80% among adolescent girls in rural Nagpur, significantly surpassing both the national average of 53.1% (NFHS-4) and the pooled estimate of 65.7% from a recent meta-analysis by Daniel et al. [09]. Although our findings align with the 88.3% prevalence reported in rural Haryana, they contrast sharply with lower estimates such as 44% in Tamil Nadu and 42.75% in Maharashtra [10-12]. These discrepancies reflect the strong influence of localized factors, including socioeconomic conditions, dietary patterns, and the effectiveness of public health interventions across regions.

Physiologically, the findings are consistent with known vulnerabilities during adolescence. Iron demands increase substantially due to rapid growth, menarche, and menstrual blood loss needs that are rarely met in settings with low intake of bioavailable iron and limited dietary diversity [13]. The clustering of moderate and severe anemia among older adolescents (17-19 years) in our study underscores this biological stress. Importantly, our findings support the view that anemia in adolescent girls is not merely a result of dietary insufficiency but a multifactorial condition shaped by physiological, educational, cultural, and health system barriers. Addressing it requires an integrated public health approach, one that couples nutrition with menstrual health education, community engagement, and strengthened service delivery.

In our study, anemia severity was significantly associated with increasing age and the presence of menstrual morbidities, while BMI did not demonstrate a statistically significant relationship. These findings are consistent with observations from rural Maharashtra, where Ahankari et al. (2017) noted that anemia risk increased with age and was inversely associated with mid-upper arm circumference and frequency of fruit consumption-underscoring the influence of physiological maturation and dietary quality [14]. Similarly, Shah et al., in a study conducted among adolescent girls in rural Maharashtra, reported that menstrual morbidities, low maternal education, and frequent intake of junk food were strongly associated with anemia [15]. These findings align closely with our results, reinforcing the role of reproductive health and maternal knowledge as key contributors to iron deficiency.

Maternal education emerged as a protective factor among the adolescent girls, finding that echoes the results from Subramanian et al. (2022) in Haryana, where girls whose mothers had higher education levels had significantly lower odds of anemia [16]. Moreover, our findings are reinforced by Yadav et al. (2023), who emphasized the role of menstrual hygiene practices such as the type and number of sanitary pads used, and the duration of menstrual flow as critical determinants of anemia risk among rural adolescent girls in Uttar Pradesh [17]. Interestingly, the non-significant association between BMI and anemia in our data diverges from studies like Goyal (2016), which found strong links between underweight status and anemia. However, this divergence aligns with a growing body of evidence suggesting that anemia can exist across a range of body compositions, possibly reflecting subclinical micronutrient deficiencies and chronic inflammatory states [18].

Collectively, these findings highlight the multifactorial nature of anemia among rural adolescent girls in India. While age and reproductive health remain biologically grounded predictors, behavioral patterns such as dietary intake and menstrual hygiene, along with social determinants like maternal education and family resources, critically shape anemia risk. The absence of a consistent link with BMI emphasizes the limitations of using anthropometric indicators alone to screen for anemia. Thus, a shift toward comprehensive risk profiling, including menstrual history, food diversity, and maternal literacy, is essential. These insights call for integrated interventions that extend beyond supplementation to holistically address the ecological determinants of adolescent health.

The findings of this study have significant public health implications, underscoring the urgent need for integrated, community-based interventions targeting adolescent girls in rural India. The prevalence of anemia (80%) and its strong associations with age, menstrual morbidities, and maternal education reveal critical gaps in both preventive and promotive health services. This calls for a paradigm shift from isolated iron supplementation efforts to comprehensive adolescent health programs that include menstrual health education, dietary counseling, and maternal literacy initiatives. Moreover, the lack of association between anemia and BMI highlights the inadequacy of current anthropometric screening tools in identifying at-risk individuals, reinforcing the need for routine hemoglobin testing and broader micronutrient surveillance at the primary care level. Addressing anemia in adolescence is not only essential for individual well-being but also crucial for breaking the intergenerational cycle of malnutrition, poor educational attainment, and adverse maternal outcomes, making it a public health priority with long-term societal impact [19].

A major strength of this study lies in its community-based design and the use of systematic random sampling, which enhances the representativeness and external validity of the findings for rural adolescent populations. The relatively large sample size, standardized data collection protocols, and the inclusion of both biological (hemoglobin, BMI) and reproductive health indicators (menstrual morbidities) further strengthen the comprehensiveness of the analysis. Additionally, the study contributes valuable primary data from a rural Indian setting, addressing a critical evidence gap often overlooked in national surveys. However, certain limitations must be acknowledged. Hemoglobin estimation was performed using Sahli’s method, which, although suitable for field settings, may lack the precision of automated hematology analyzers, particularly for detecting mild anemia. The cross-sectional nature of the study precludes causal inference, and reliance on self-reported menstrual characteristics may introduce recall and reporting bias. Despite these limitations, the study provides robust insights that are highly relevant for informing adolescent health interventions in underserved regions.

## Conclusions

This study demonstrates a disproportionately high burden of anemia among rural adolescent girls in Nagpur district, Maharashtra, India, with 80% of participants affected and over one-third experiencing moderate to severe forms. The significant associations between anemia severity, advancing adolescent age, and menstrual morbidities highlight the need for early, targeted interventions that extend beyond nutritional supplementation alone. Based on these findings, it is recommended to have the integration of routine hemoglobin screening and menstrual health assessments into adolescent health outreach programs such as RKSK and WIFS, particularly for girls aged 14 years and above. Training frontline health workers to recognize and manage menstrual abnormalities and expanding community-based iron and folic acid supplementation coverage are essential. Furthermore, school-based health education curricula should incorporate menstrual health literacy and anemia awareness to promote timely health-seeking behaviour and dietary changes before anemia becomes clinically severe.
